# Supplementary material for: Alpha synuclein modulates mitochondrial Ca2+ uptake from ER during cell stimulation and under stress conditions
Source: NPJ Parkinsons Dis. 2023 Sep 23;9:137. doi: 10.1038/s41531-023-00578-x (PMC10518018; doi:10.1038/s41531-023-00578-x)
Supplement: Supplementary file 1 — Supplementary Information [file 41531_2023_578_MOESM1_ESM.pdf]

Alpha Synuclein Modulates Mitochondrial Ca^{2+} Uptake from ER During Cell Stimulation and Under Stress Conditions

SUPPLEMENTARY FIGURES

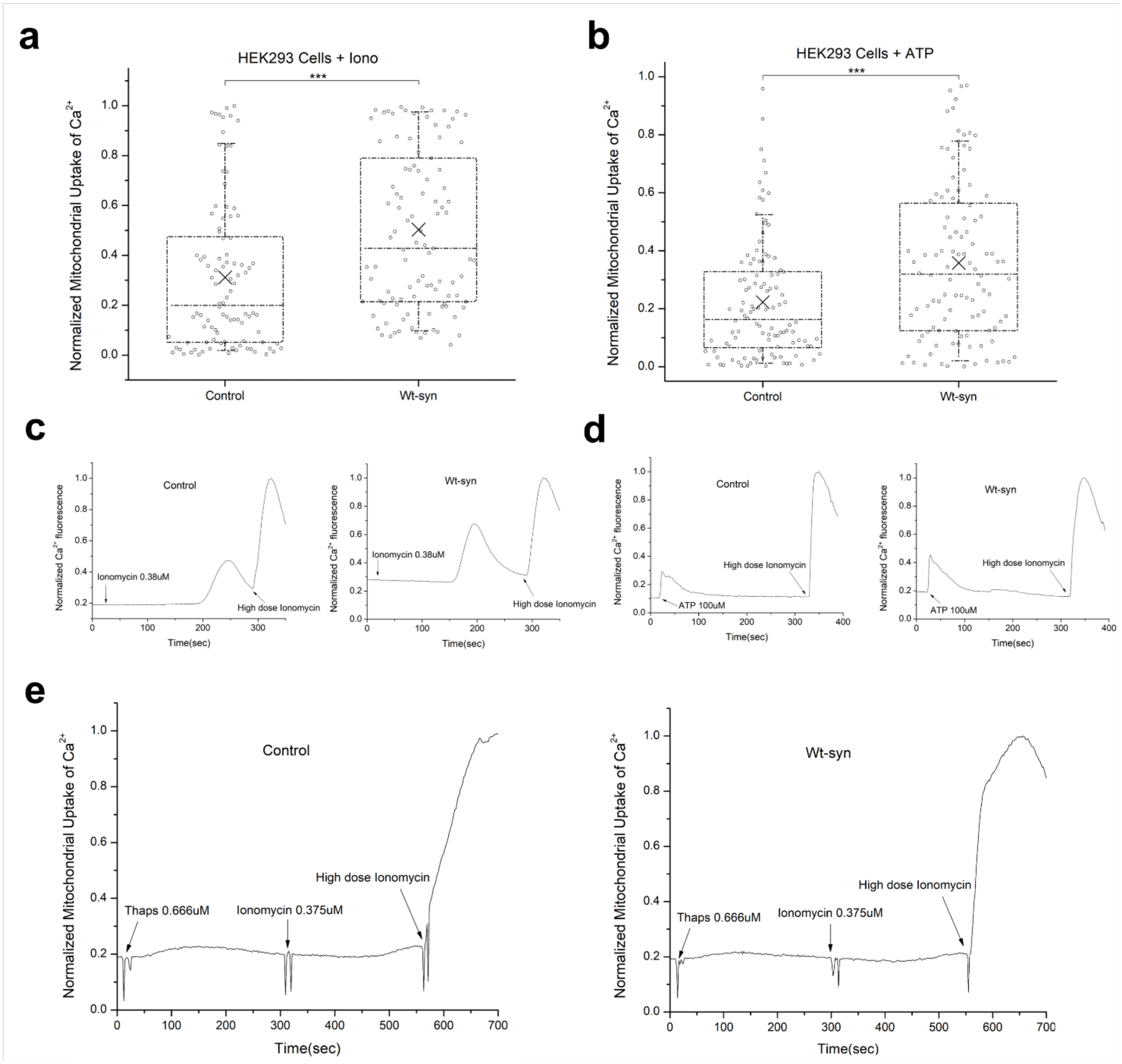

Supplementary Figure 1. Wt-syn enhances mitochondrial Ca^{2+} uptake from ER in HEK293 cell as stimulated by ionomycin or ATP. Comparative stimulation data and representative traces for experiments shown in Figure 3a of main text. HEK293 cells were co-transfected with plasmids for mito-GCaMP6f and mRFP (control) or Wt-syn (via multicistronic construct Syn-p2a-mRFP). Cells were imaged by confocal microscopy, and (comparable) expression levels of Wt-syn under these transfection conditions were determined using mRFP fluorescence (expressed by the same multicistronic construct). Mitochondrial Ca^{2+} uptake was stimulated by low-dose ionomycin (0.38 µM, *a, c, e*) or ATP (100 µM, *b, d*) without (*a - d*) or with (*e*) pre-treatment with thapsigargin (0.67 mM). Mito-GCaMP6f fluorescence was monitored in confocal movies before and after stimulation, and after indicated addition of high-dose ionomycin (5 µM, 300-400 sec later) to achieve the maximal fluorescence value for normalization. ***a, b***) Mitochondrial Ca^{2+} uptake stimulated by ionomycin (*a*) or ATP (*b*) for individual cells using Eq 2. Each sample comprising n~80 co-transfected cells are from three independent experiments. The box shows 25th-75th percentile of the data, midline shows median, and X shows average; *** represents P-values <0.001. The data shown in *a* of this figure are the same as those shown in Figure 3*a* (solid outlines, -Thaps). ***c, d, e***) Representative traces of mito-GCaMP6f fluorescence integrated over 5-7 cells within confocal fields. Arrows indicate addition of thapsigargin (0.67 mM) (*e*), low-dose ionomycin (*c, e*) or ATP (*d*) and high-dose ionomycin (*c, d, e*) for control cells and cells expressing Wt-syn.

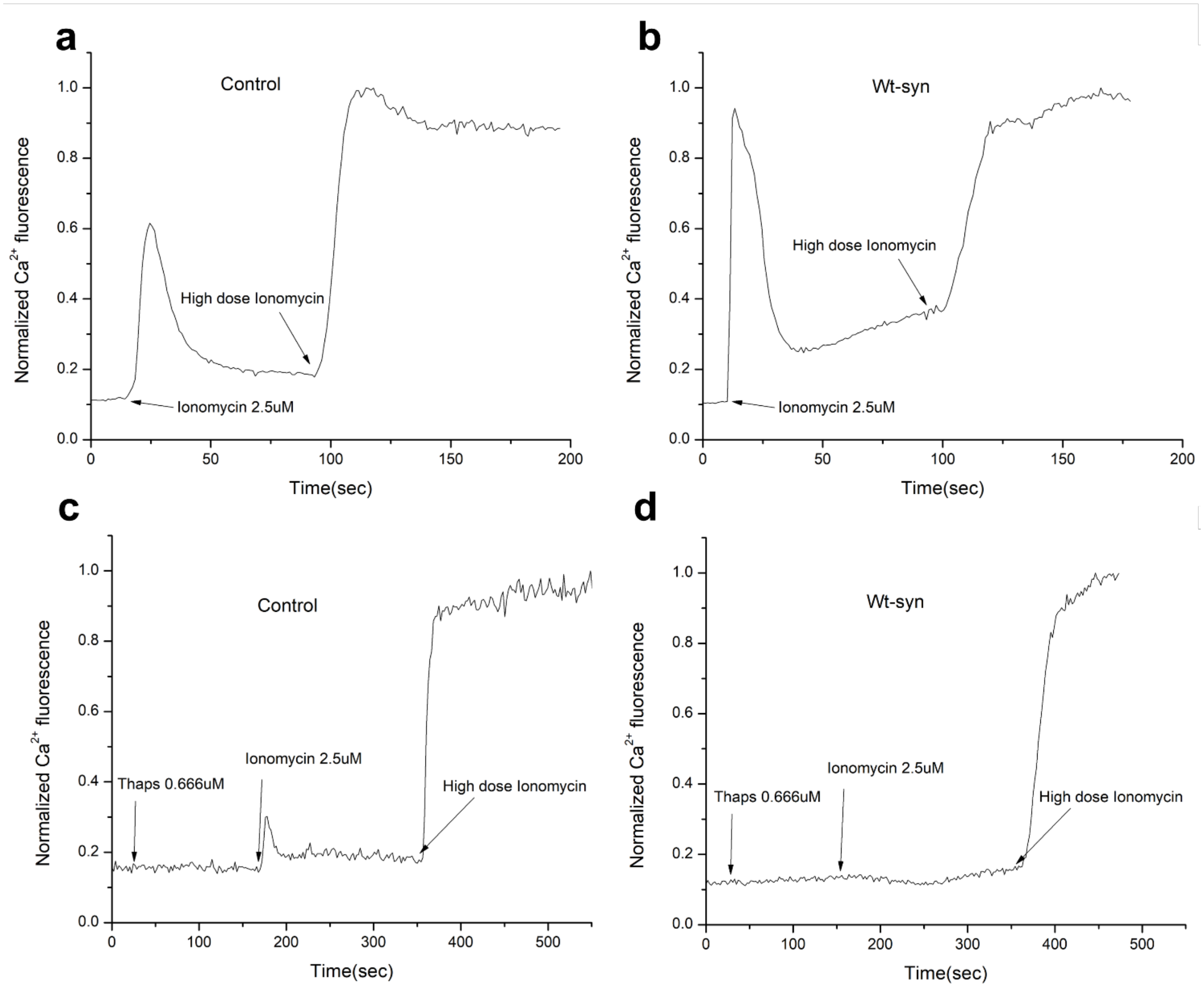

Supplementary Figure 2. Wt a-syn enhances mitochondrial Ca^{2+} uptake from ER in dopaminergic N2a cells. ***a, b, c, d***) Representative traces for experiments shown in Figure 3b of main text. Dopaminergic N2a cells were co-transfected with mito-GCaMP6f and mRFP (control) (*a, c*) or Wt-syn (via multicistronic construct Syn-p2a-mRFP) (*b, d*). Cells were imaged by confocal microscopy, and (comparable) expression levels of Wt-syn under these transfection conditions were determined using mRFP fluorescence (expressed by the same multicistronic construct). Cells were stimulated by low-dose ionomycin (2.5 μM) without (*a, b*) or with (c, d) pre-treatment with thapsigargin (0.67 mM). Mito-GCaMP6f fluorescence was monitored in confocal movies before and after thapsigargin and stimulation, and after addition of high-dose Ionomycin (8 μM, 200-300 sec later) to achieve maximal fluorescence value. Traces integrated from confocal fields containing 1-3 cells.

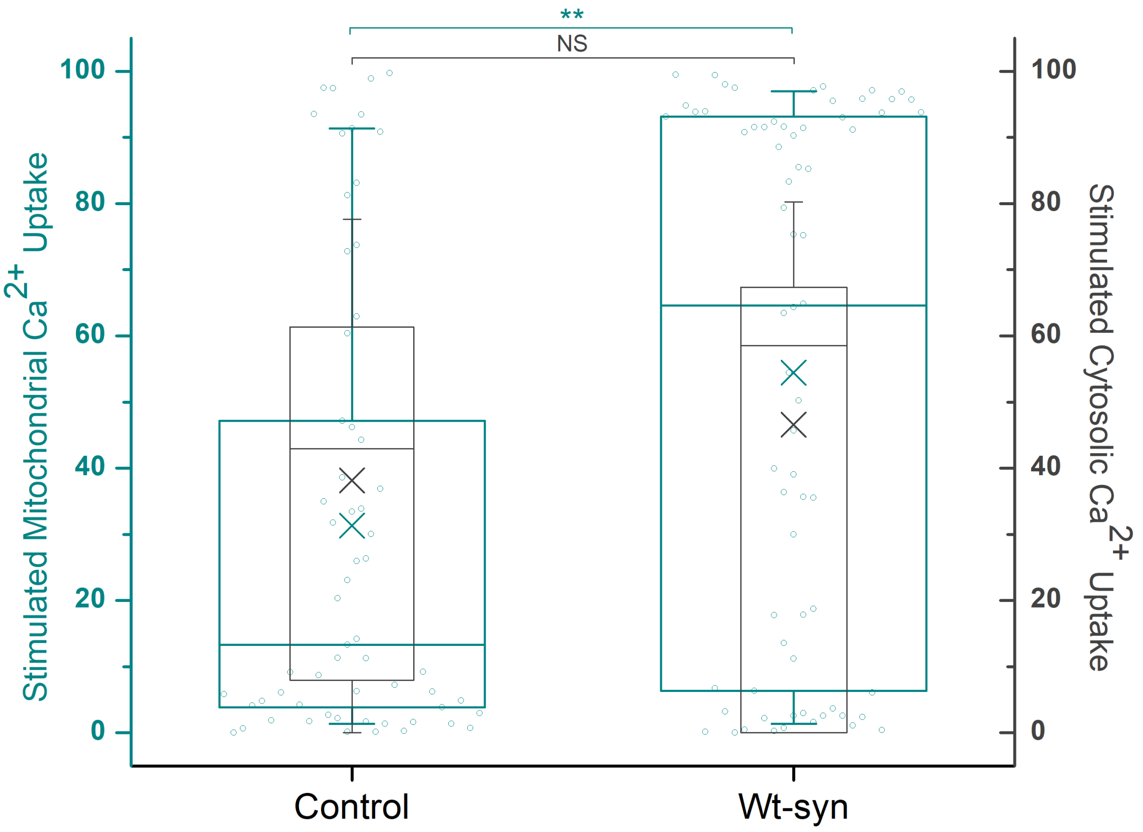

Supplementary Figure 3. Enhancement of stimulated mitochondrial Ca^{2+} uptake by Wt-syn is not due to increased cytosolic Ca^{2+} uptake. RBL cells were co-transfected with plasmids for either mito-jRCaMP1b (mitochondrial Ca^{2+} indicator), GCaMP3 (Cytosolic Ca^{2+} indicator) and one of pcDNA (empty vector control) or Wt-syn (in pcDNA vector). Harvested cells were transferred to Ca^{2+}-free BSS buffer containing 2 μM EGTA, then imaged by confocal microscopy; Wt-syn expression levels (in the same range as for other experiments) were determined with anti α,β-synuclein (1:150 dilution). Mitochondrial Ca^{2+} (left axis) and cytosolic Ca^{2+} (right axis) were monitored in confocal movies before and after stimulation by antigen, and after indicated addition of high-dose ionomycin (5 μM, 300-400 sec later) to achieve maximal fluorescence for each Ca^{2+} indicator. Normalized data points shown are stimulated mitochondrial Ca^{2+} uptake (Eq 2). Normalized data for both mitochondrial (green, left) and cytosolic (black, right) Ca^{2+} increases are represented in superimposed box plots. Each sample comprising n~60 cells are from three independent experiments. The box shows 25th-75th percentile of the data, midline shows median, and X shows average. Error bars are ± SEM; ** represents P-values <0.01, NS represents not significant (P-values > 0.05).

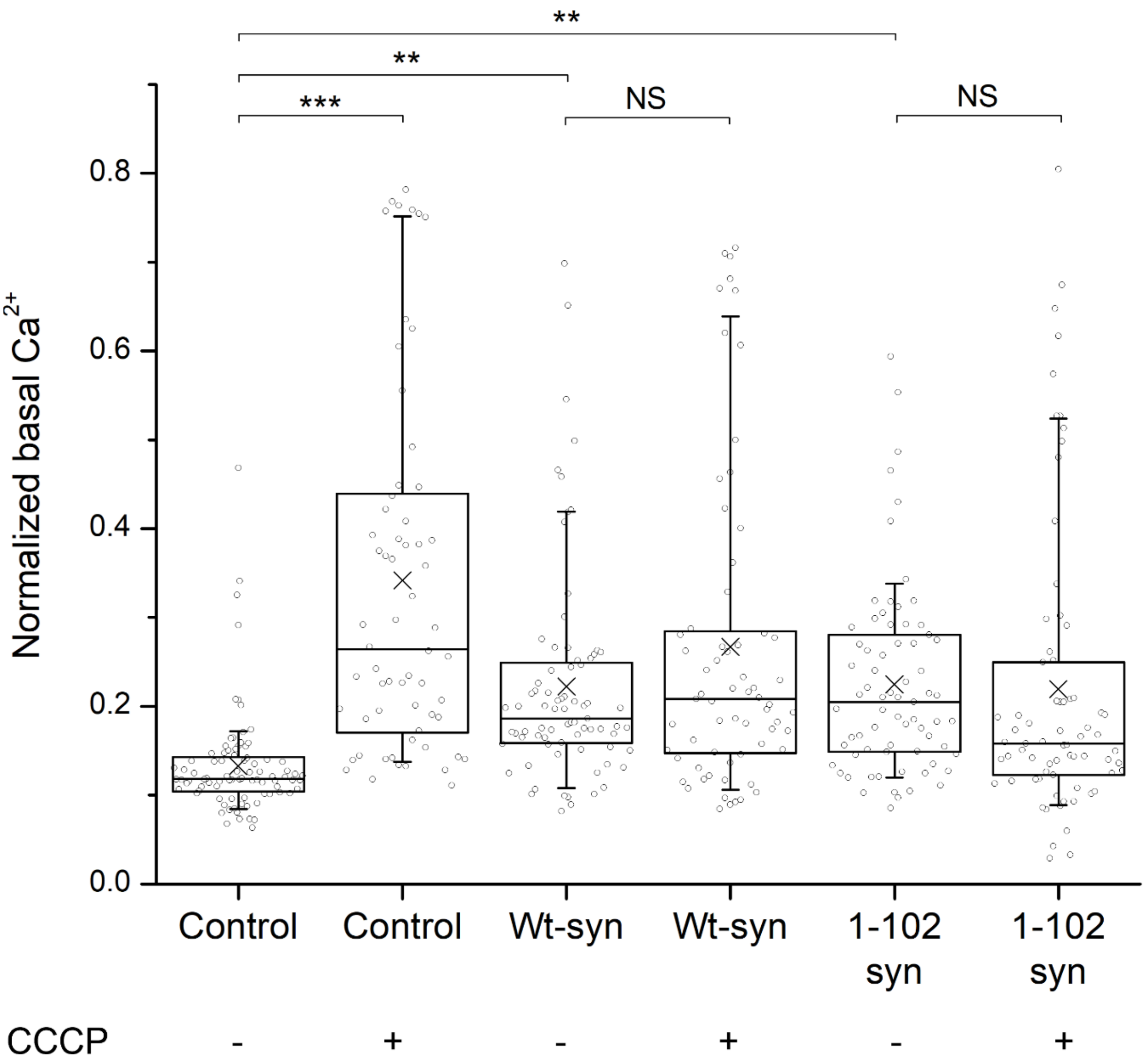

Supplementary Figure 4. Expression of Wt-syn and 1-102-syn enhances resting level of mitochondrial Ca^{2+}. In same experiment as Figure 5a, RBL cells were co-transfected with plasmids for mito-GCaMP6f and one of syn-p2a-mRFP, mRFP (control), or 1-102-syn (in pcDNA vector). Cells were imaged by confocal microscopy, and (comparable) expression levels of Wt-syn under these transfection conditions was determined using mRFP fluorescence (expressed by the same multicistronic construct) or co-transfected mito-GCaMP6f fluorescence (see Figure S6*b, c, d*). Samples were treated or not with 10μM CCCP for 30 min, then washed with RBL media and incubated in media for 3 hours (recovery), followed by imaging using confocal microscopy. Mito-GCaMP6f fluorescence was monitored in confocal movies initially and after addition of high-dose ionomycin (5 μM) to achieve maximal fluorescence. Each sample comprising n~100 co-transfected cells are from three independent experiments. Data points were calculated using Eq 3. The box plots show 25th-75th percentile of the data; midline shows median, and X shows average; ** represents P-values <0.01, *** represents P-values <0.001. NS represents not significant (P-values > 0.05).

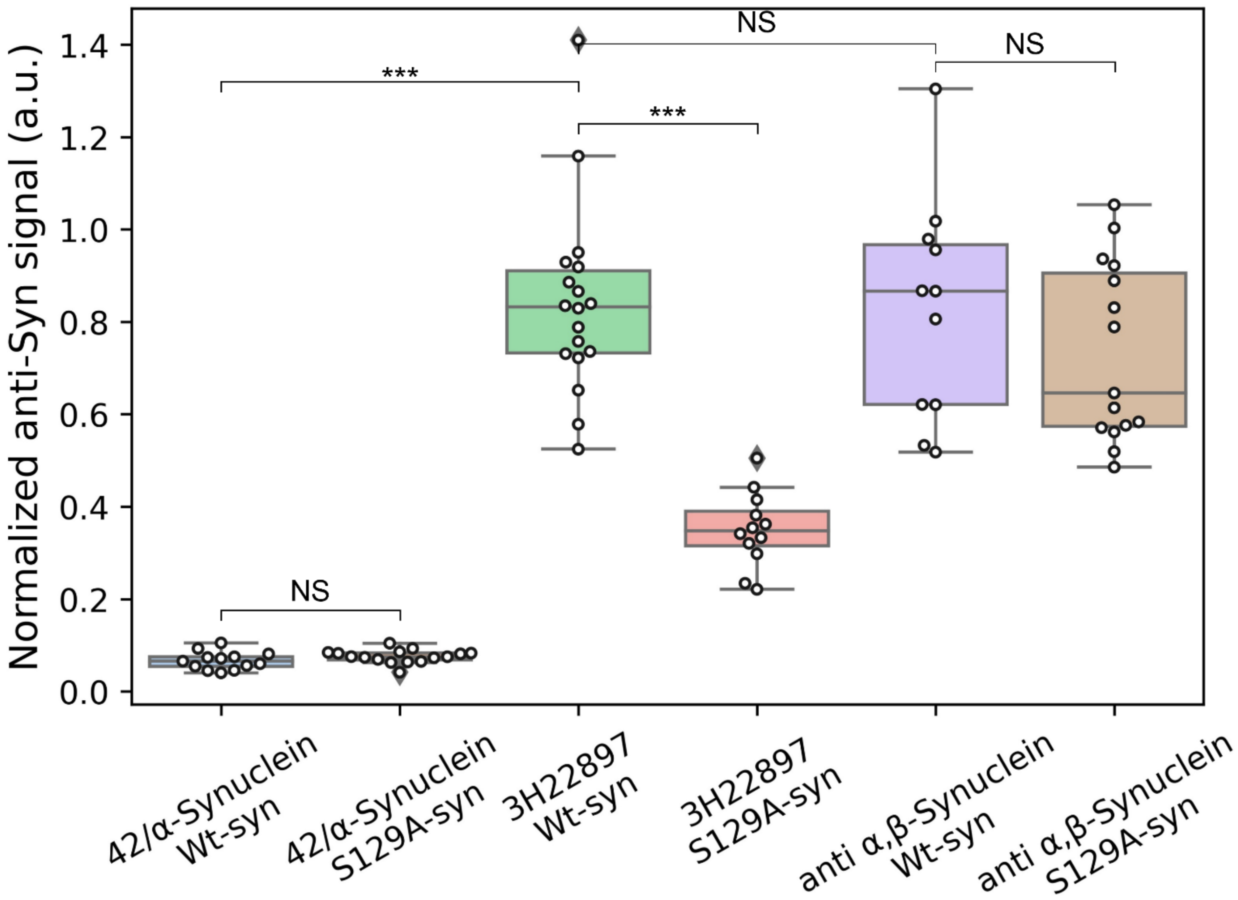

<u>Supplementary Figure 5. Comparison of Anti-syn antibodies for binding to Wt-syn and S129A-syn.</u> RBL cells were transfected with 10 μg of multicistronic vectors syn-p2a-mRFP or S129A-syn-p2a-mRFP, following standard protocols (see Methods). Fixed cells were labeled with, as indicated, anti α,β-synuclein (DHSB, 1:150 dilution), 42/α-Synuclein (BD Biosciences, 1:200 dilution) or 3H2897 (Santa Cruz Biotechnology, 1:200 dilution), followed by a secondary antibody conjugated to Alexa Fluor 488, and then imaged by confocal microscopy. Immunostained images were collected and quantified following our standard protocols. 10 – 15 cells for each sample were evaluated. The anti-syn signal (Alexa Fluor 488) was normalized against the mRFP signal from syn-p2a-mRFP to account for the cell by cell variation of a-syn expression. The box plots show 25th-75th percentile of the data; midline shows median;*** represents P-values <0.001. NS represents not significant (P-values > 0.05).

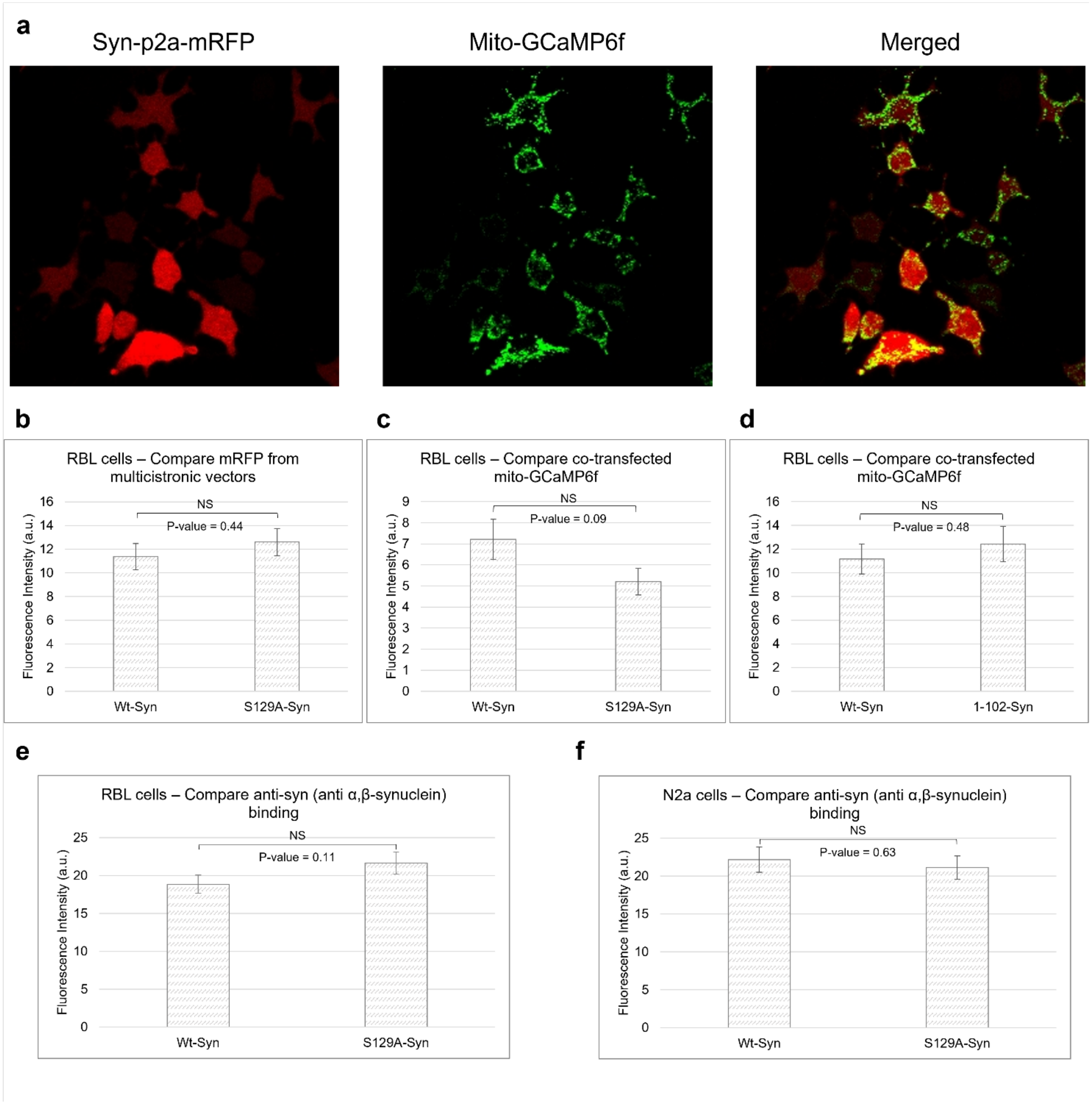

Supplementary Figure 6. Confirmation of similar expression levels of a-syn variants using corresponding fluorescent markers. Because comparing functional effects of different variables requires that expression levels of a-syn variants be in the same range, transfection conditions and other experimental procedures were developed to ensure this outcome as described in Methods. Confirmation of similar expression levels is demonstrated in the following representative examples. Quantification of confocal images followed our standard protocol (see Methods). ***a-d***) RBL cells were co-transfected with plasmids for mito-GCaMP6f and one of syn-p2a-mRFP, S129A-syn-p2a-mRFP, mRFP (control), or 1-102-syn in pcDNA vector. Data were taken from the experiment of Figure 5*a*. ***a***) Confocal images of mRFP (red) co-expressed with Wt-syn in syn-p2a-mRFP and co-

transfected mito-GCaMP6f (green), showing proportional co-transfection. ***b***) Expression of Wt-syn compared to S129A-syn as determined with mRFP expressed by the same multicistronic construct. ***c***) Expression of Wt-syn compared to S129A-syn as determined with co-transfected mito-GCaMP6f. ***d***) Expression of Wt-syn compared to 1-102-syn as determined with co-transfected mito-GCaMP6f. ***e***) RBL cells or ***f***) N2a cells were transfected with Wt-syn or S129A-syn in pcDNA vectors. Data (without CCCP condition) were taken from experiment of Figure 7*c, f* (RBL cells), and *g, h* (N2a cells). Expression of Wt-syn compared to S129A-syn as determined with immunostaining with anti α,β-synuclein (1:150 dilution) following our standard protocols. 70-80 cells for each sample were evaluated; Error bars represent SEM; NS represents not significant (P-values > 0.05).

SUPPLEMENTARY MOVIES LEGENDS

Supplementary Movie 1. RBL cells transfected with Wt-syn exhibit high level of mitochondrial Ca^{2+} uptake when stimulated with sub-optimal concentration of antigen. Movie corresponds to trace shown in Figure 2b of main text; image field includes about 25 cells. RBL cells were co-transfected with plasmids for mito-GCaMP6f and Wt-syn (pcDNA) and imaged by confocal microscopy. Mitochondrial Ca^{2+} uptake was monitored mito-GCaMP6f fluorescence before (starting at -16 sec) and after stimulation by Ag (DNP-BSA, 1 ng/ml, starting at 0 sec), followed by addition of high-dose ionomycin (5µM, starting at 370 sec).

Supplementary Movie 2. RBL cells transfected with empty vector exhibit little mitochondrial Ca^{2+} uptake when stimulated with sub-optimal concentration of antigen. Movie corresponds to trace shown in Figure 2c of main text; image field includes about 25 cells. RBL cells were co-transfected with plasmids for mito-GCaMP6f and pcDNA (empty vector control) and imaged by confocal microscopy. Mitochondrial Ca^{2+} uptake was monitored mito-GCaMP6f fluorescence before (starting at -16 sec) and after stimulation by Ag (DNP-BSA, 1 ng/ml, starting at 0 sec), followed by addition of high-dose ionomycin (5µM, starting at 370 sec).
